# Hybrid two-mode squeezing of microwave and optical fields using optically pumped graphene layers

**DOI:** 10.1038/s41598-020-73363-y

**Published:** 2020-10-07

**Authors:** Montasir Qasymeh, Hichem Eleuch

**Affiliations:** 1 Electrical and Computer Engineering Department, Abu Dhabi University, Abu Dhabi, UAE; 2 Department of Applied Physics and Astronomy, University of Sharjah, Sharjah, UAE; 3 Institute for Quantum Science and Engineering, Texas AM University, Texas, USA

**Keywords:** Quantum optics, Electrical and electronic engineering

## Abstract

A measurable quadrature of a squeezed quantum state manifests a small uncertainty below the Heisenberg limit. This phenomenon has the potential to enable several extraordinary applications in quantum information, metrology and sensing, and other fields. Several techniques have been implemented to realize squeezed electromagnetic states, including microwave fields and optical fields. However, hybrid squeezed modes (that incorporate both microwave and optical fields) have not yet been proposed despite their vital functionality to combine the two worlds of quantum superconducting systems and photonics systems. In this work, for the first time, we propose a novel approach to achieve two-mode squeezing of microwave and optical fields using graphene based structure. The proposed scheme includes a graphene layered structure that is driven by a quantum microwave voltage and subjected to two optical fields of distinct frequencies. By setting the optical frequency spacing equal to the microwave frequency, an interaction occurs between the optical and microwave fields through electrical modulation of the graphene conductivity. We show that significant hybrid two-mode squeezing, that includes one microwave field and one optical field, can be achieved. Furthermore, the microwave frequency can be tuned over a vast range by modifying the operation parameters.

## Introduction

Microwave fields with squeezed states hold promises for realizing quantum communication systems^1^ and fault-tolerant quantum computation^2^ and for connecting quantum computers^3^. Additionally, such fields can enable many unprecedented applications, including quantum radar and navigation^4–6^, quantum metrology^7^, and weak classical signal detection^8^. Moreover, squeezed optical fields, which are equally functional to all above applications, are also used in gravitational wave detection^9^, laser system stabilization^10^, achieving accurate gyroscope systems^11^, detecting single-molecule^12^, and to realize quantum memory^13^, just to mention few.

Mainly three configurations have been successfully implemented to achieved squeezed microwave fields. These are Josephson parametric amplifiers (JPAs)^14^, superconductor resonators^15^, and electromechanical resonators^16^. Microwave squeezing with JPAs is based on using the JPA nonlinear response to form nonlinear resonators^17^. A typical squeezing gain of approximately 10 dB over a few MHz bandwidth is achieved^18^. Extended designs including Josephson traveling wave amplifiers with a squeezing gain of 20 dB and a bandwidth up to a few GHz have also been reported^19^. However, phase matching is required^20^. In contrast, microwave squeezing with gain up to 8 dB is achieved using superconductor resonators by implementing dissipation engineering to a coupled microwave field^21,22^. Squeezing with electromechanical resonators has been reported by using the radiation pressure force of the interacting field^23^. Squeezing gains up to 8 dB over a few tens of MHz are typically achieved^24^. However, the operation is temperature dependent, and the performance degrades for higher microwave frequencies. Similarly, squeezed optical fields have been achieved with gain of more than 15 dB either by implementing optical nonlinear materials^25^ or by incorporating optomechanical systems^26^. Optical squeezing utilizing nonlinear optical materials are conducted by means of wave mixing or parametric down-conversion^27^, while optical squeezing utilizing optomechanical systems is realized by coupling the light photons to mechanical motion via incorporating mechanical resonator in an optical cavity^28^.

In this work, we propose a novel scheme for hybrid two-mode squeezing of microwave and optical fields. The proposed scheme utilizes an electro-optic interaction obtained by electrically modulating the graphene conductivity. A microwave field (of frequency $\omega _m$) drives the graphene layers. The graphene layers are subject to two optical fields with frequencies $\omega _1$ and $\omega _2$. The interaction between the microwave and optical fields is enabled by setting $\omega _1-\omega _2=\omega _m$. A quantum mechanics model is developed to describe the electro-optic interaction. The microwave and optical fields are determined in the steady state, in which the time rate changes of their averages are zero. The operator fluctuations are evaluated by calculating the squeezing spectrum. The microwave field is conceived as the signal, while the optical fields at $\omega _1$ and $\omega _2$ are considered as the pump and the idler, respectively. We show that hybrid two-mode squeezing—that includes microwave field (i.e., the signal) and optical field (i.e., the idler)—can be achieved with a peak squeezing gain of 36 dB over about 2 MHz fluctuation spectrum bandwidth. Furthermore, the squeezed microwave frequency can be tuned over a wide range by modifying the optical frequency spacing. Achieving a hybrid two-mode squeezing paves the way towards merging the superconducting quantum systems with photonics systems. It then ultimately leads to hybrid systems that leverage the advantages of both^29^. Furthermore, the advantages of our proposed squeezing scheme include its simple structure (with no phase matching or SQUID insertion required), moderate cryogenic operation temperature, and tunability over a vast microwave frequency range.

## Results

### Model

**Figure 1:**
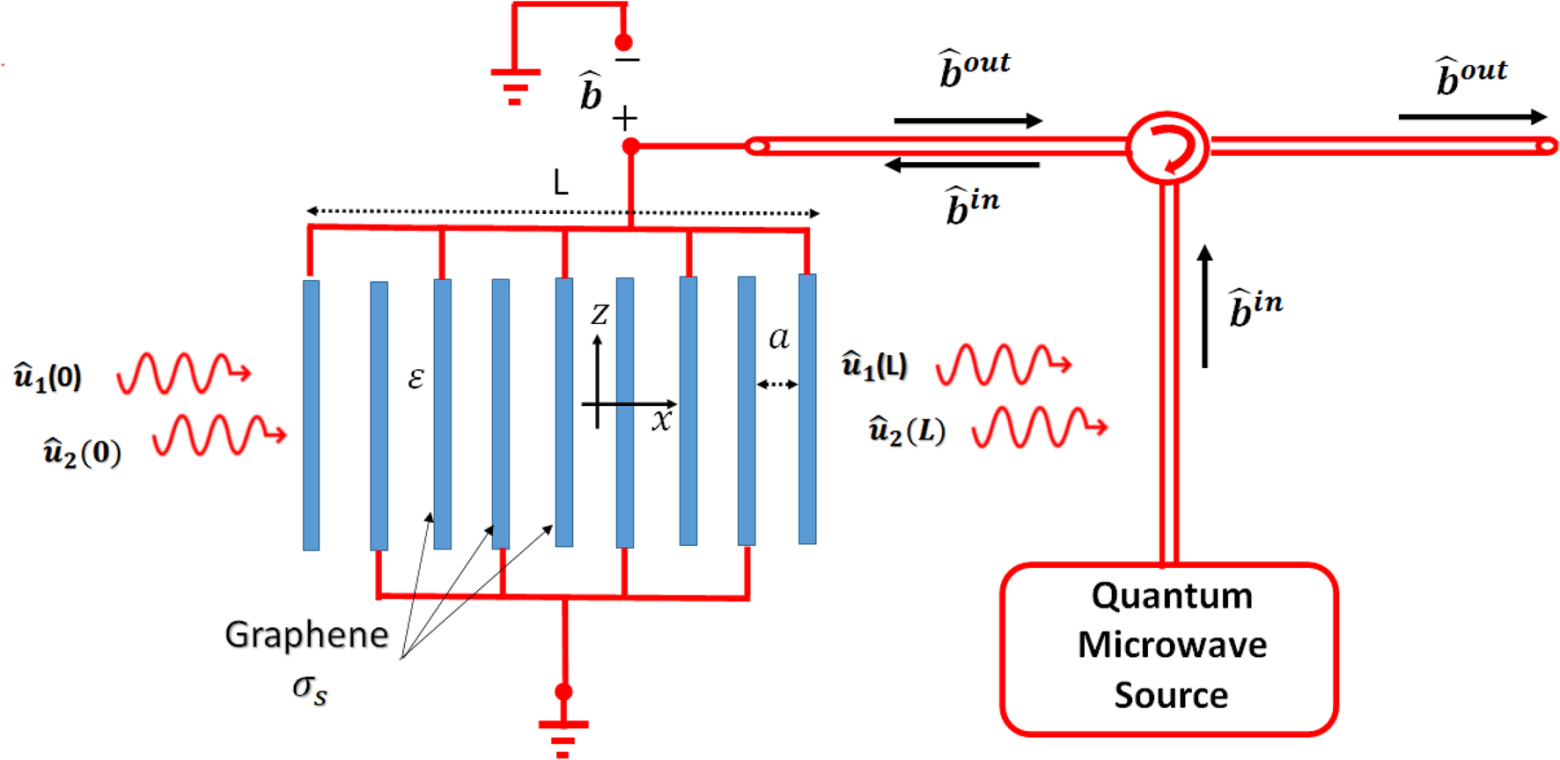
Proposed graphene layered structure driven by a ${\hat{b}}$ microwave field and subject to ${\hat{u}}_1$ and ${\hat{u}}_2$ optical input fields.

**Figure 2:**
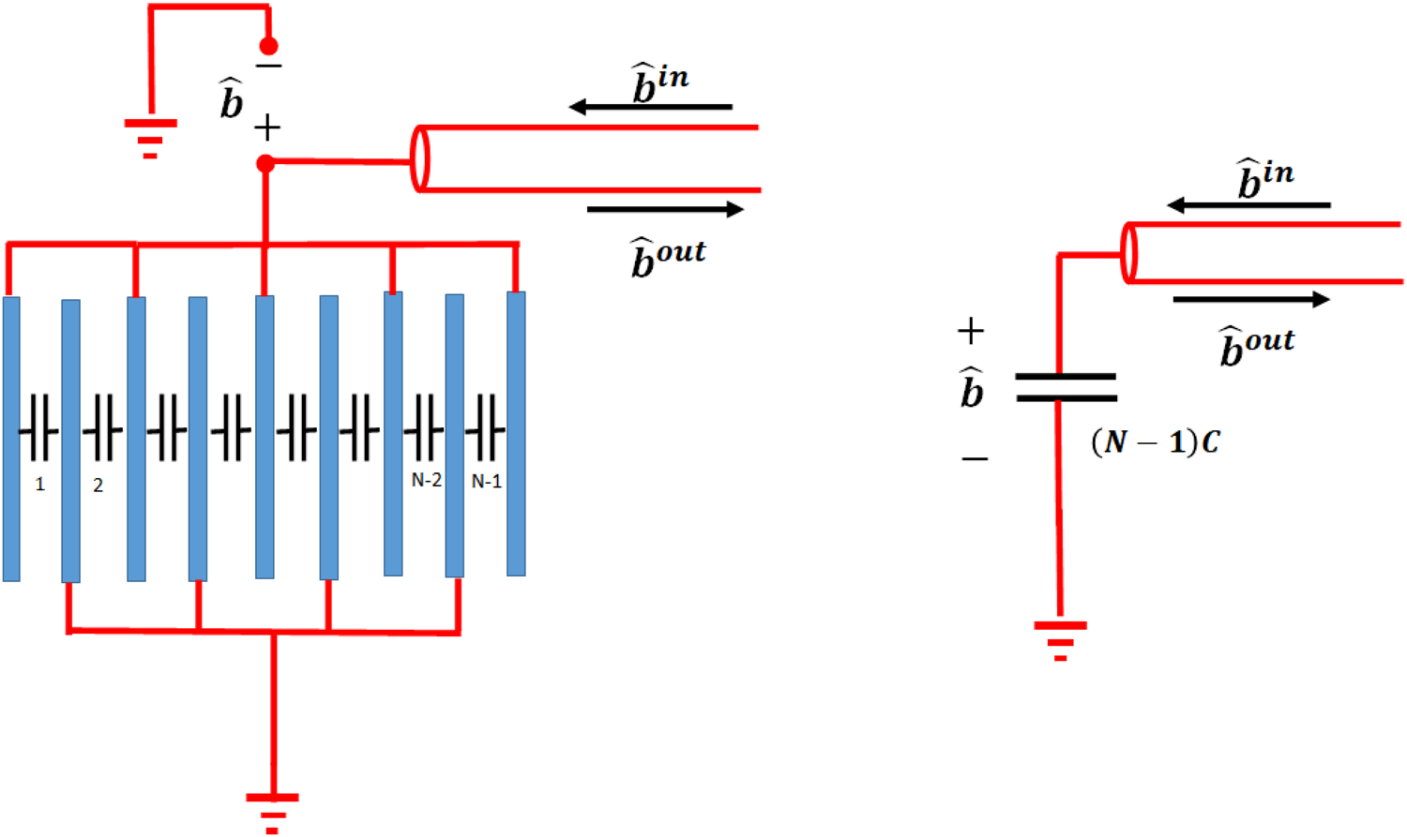
Equivalent electrical circuit of the graphene layer structure.

The proposed structure is composed of periodic graphene layers connected in an interdigital configuration and electrically driven by a transmission line, as shown in Fig. 1. The graphene layers have periodicity *a* and cross-sectional area $A_r$ and are filled with a dielectric material of permittivity $\varepsilon$. The transmission line is used to electrically drive the graphene layers by a microwave ${\hat{b}}^{in}$ voltage operator of frequency $\omega _m$. The transmission line is connected to every other graphene layer, while the middling graphene layers are grounded. Thus, the graphene layers can be considered as $(N-1)$ identical shunted capacitors, each of $C=\frac{\varepsilon \varepsilon _0 }{a}$ per unit area capacitance^30^. Electrically, the graphene layers are equivalent to a capacitor of $C_T=(N-1)C$ per unit area capacitance, as shown in Fig. 2. Here, *N* is the number of graphene layers. In this work, the intrinsic impedance of the transmission line is considered to be much smaller than the capacitor impedance of the graphene layers, yielding total microwave reflection. Thus, the total microwave voltage driving the graphene layers is given by ${\hat{b}}={\hat{b}}^{out}+{\hat{b}}^{in}$, where ${\hat{b}}^{out}$ and ${\hat{b}}^{in}$ are the reflected and incident microwave voltage operators, respectively.

Additionally, two optical fields ${\hat{u}}_1$ and ${\hat{u}}_2$ are launched normally to the graphene layers. The optical fields have distinct frequencies $\omega _1$ and $\omega _2$, respectively. By setting $\omega _1-\omega _2=\omega _m$, interaction of the optical and microwave operators is enabled by electrically controlling the graphene conductivity. Optically, the graphene layers can be described by the concept of the effective permittivity, which can be obtained from the dispersion relation $\cos (a \beta )=\cos \bigg (a \sqrt{\varepsilon }\frac{\omega }{c}\bigg )-i\frac{Z_0}{2\sqrt{\varepsilon }} \sin \bigg (a \sqrt{\varepsilon }\frac{\omega }{c}\bigg )\sigma _s$^31^. Here, $\beta$ represents the optical propagation constant, $Z_0=377~\Omega$ is the free space impedance, *c* denotes the speed of light in vacuum, $\sigma _s= \frac{iq^2}{4\pi \hbar }ln\bigg (\frac{2\mu _c-(\frac{\omega }{2\pi }+i\tau ^{-1})\hbar }{2\mu _c+(\frac{\omega }{2\pi }+i\tau ^{-1})\hbar }\bigg )+\frac{iq^2 K_B T}{\pi \hbar ^2(\frac{\omega }{2\pi }+i\tau ^{-1})}\bigg (\frac{\mu _c}{K_B T}+2 ln \big ( e^{-\frac{\mu _c}{K_B T}}+1\big ) \bigg )$ is the graphene conductivity, *q* represents the electron charge, $\hbar$ denotes Planck’s constant, $\tau$ is the scattering relaxation time in graphene, $K_B$ represents the Boltzmann constant, and *T* is the temperature.

Following a similar perturbation approach to that developed in our previous work (see Supplementary Material 1)^30,32^, the quantum Hamiltonian $\hat{{\mathcal {H}}} = \hat{{\mathcal {H}}_0}+\hat{{\mathcal {H}}_1}$ is given by:

1 $$\begin{aligned} \hat{{\mathcal {H}}_0}= & {} \hbar \omega _m {\hat{b}}^\dagger {\hat{b}}+\sum _{j=1}^{2} \hbar \omega _j {\hat{u}}_{j}^\dagger {\hat{u}}_j, \end{aligned}$$

2 $$\begin{aligned} \hat{{\mathcal {H}}_1}= & {} \hbar g {\hat{u}}_{2}^\dagger {\hat{b}}^\dagger {\hat{u}}_{1} +\hbar g^* {\hat{u}}_{1}^\dagger {\hat{b}} {\hat{u}}_{2} +h.c., \end{aligned}$$

where $\hat{{\mathcal {H}}_0}$ and $\hat{{\mathcal {H}}_1}$ are the free and interaction Hamiltonians, respectively, $g= \varepsilon ^{\prime \prime } \sqrt{\frac{\omega _{1} \omega _{2}}{\varepsilon _{1}^{\prime }\varepsilon _{2}^{\prime }}}\sqrt{\frac{ \hbar \omega _m}{C A_r}} sinc \big ( \frac{\beta _{1}-\beta _{2}}{2} L\big ) e^{i\frac{\beta _{1}-\beta _{2}}{2}L}$ is the coupling rate, *h*.*c*. denotes the Hermitian conjugate, $\varepsilon ^{\prime } =\frac{\beta ^2}{k_0^2}$ is the effective permittivity, $\varepsilon ^{\prime \prime } =2\frac{\beta \beta ^{\prime \prime }}{k_0^2}$ represents the permittivity perturbation, $\beta ^{\prime \prime }= i\frac{Z_0}{2 d \sqrt{\varepsilon }}\frac{ \sin \bigg (d \frac{2 \pi f \sqrt{\varepsilon }}{c}\bigg )}{\sin (d\beta )} \sigma _{s}^{\prime \prime }$ is the propagation constant perturbation, $\sigma _{s}^{\prime \prime }=\frac{iq^2}{\pi \hbar }\frac{(f+i\tau ^{-1})\hbar }{4(\mu _{c})^2-(f+i\tau ^{-1})^2\hbar ^2}\mu _{c}^{\prime \prime }+\frac{iq^2 K_B T}{\pi \hbar ^2(f+i\tau ^{-1})} tanh\bigg (\frac{\mu _{c}}{2K_B T}\bigg ) \frac{\mu _{c}^{\prime \prime }}{K_B T}$ represents the graphene conductivity perturbation, $\mu _{c}=\hbar V_f\sqrt{\pi n_0}$ denotes the graphene chemical potential, $\mu _{c}^{\prime \prime }=\hbar V_f\frac{C_T}{q \sqrt{\pi n_0}}$ is the chemical potential perturbation, and $n_0$ represents the electron density. The equations of motion can obtained by substituting the Hamiltonian into the Heisenberg equation of motion, that is, $\frac{\partial {\hat{o}}}{\partial t}=\frac{i}{\hbar } [\hat{{\mathcal {H}}},{\hat{o}}]$. Losses can be taken in to account by incorporating the decay coefficients in the equations of motion. The optical decay rate includes the mode attenuation and layers transmittance, while the microwave field decay rate can be derived from electrical dissipation^30^. Importantly, according to the fluctuation-dissipation theorem, the Langevin forces ( which represent the noise in the microwave and optical fields as the feed-back of the environment to the system) need to be included. Consequently, by implementing the standard rotation approximation, the equations of motion are given by:

3 $$\begin{aligned} \frac{\partial {\hat{b}}}{\partial t}= & {} -\frac{\Gamma _m}{2} {\hat{b}}-i g ({\hat{u}}_{1}+\alpha _1){\hat{u}}_2^{\dagger }+\sqrt{\Gamma _m}{\hat{n}}_m, \end{aligned}$$

4 $$\begin{aligned} \frac{\partial {\hat{u}}_1}{\partial t}= & {} -\frac{\Gamma _1}{2} {\hat{u}}_{1}-i g^*{\hat{b}}{\hat{u}}_2+\sqrt{\Gamma _1}{\hat{n}}_1, \end{aligned}$$

5 $$\begin{aligned} \frac{\partial \alpha _1}{\partial t}= & {} -\frac{\Gamma _1}{2} \alpha _1, \end{aligned}$$

6 $$\begin{aligned} \frac{\partial {\hat{u}}_2}{\partial t}= & {} -\frac{\Gamma _2}{2} {\hat{u}}_{2}-i g({\hat{u}}_1+\alpha _1){\hat{b}}^{\dagger }+\sqrt{\Gamma _2}{\hat{n}}_2, \end{aligned}$$

where $\alpha _1$ is the classical component of the optical mode at frequency $\omega _1$, $\Gamma _j= 2 v_g Im{(\beta _j)}+\frac{v_g}{a}ln(\frac{1}{T_0^2})$ and $\Gamma _m=-\frac{2}{t_0}ln\Big (1-\frac{\nu t_0}{2 q R_g}\Big )$ are the optical and microwave decay coefficients, respectively, ${\hat{n}}_j$ denotes the quantum Langevin noise operator, $T_0$ indicates the medium transmittance (calculated using the transfer matrix method^33^), $t_0$ is the time of flight over a single layer block, $R_g=Re(\frac{1}{\sigma _s})$, and $\nu =\bigg ( \frac{\hbar \omega _m}{ C_T A_r}\bigg )^{\frac{1}{2}} {\hat{b}}$ describes the classical counterpart of the quantum microwave annihilation operator. The quantum Langevin noise operator obeys the relations $[{\hat{n}}_j(t_1),{\hat{n}}_j(t_2)^\dagger ]=\delta (t_1-t_2)$ and $\langle n(t_1)^\dagger n(t_2) \rangle = \frac{1}{exp(\hbar \omega /k_BT)-1} \delta (t_1-t_2)$. We remark here that the optical mode at frequency $\omega _1$ is composed of quantum component and classical component. This is similar to having a strong pump component and weak field component. Analytically, this is taken into account by substituted ${\hat{u}}_1+\alpha _1$ instead of ${\hat{u}}_1$ in the Heisenberg equation to achieve Eqs. (3)–(6). The classical component $\alpha _1$ undertakes the role of the optical pumping.

By expressing the interacting operators in terms of their averages and fluctuations, i.e., ${\hat{o}}=\left\langle {\hat{o}}\right\rangle + \delta {\hat{o}}$, and then substituting them into the equations of motion in Eqs. (3)–(6), we obtain a set of rate equations for the operator averages and another set for the fluctuations^23^. The rate equations for the averages are given by:

7 $$\begin{aligned} \frac{\partial \left\langle {\hat{b}}\right\rangle }{\partial t}= & {} -\frac{\Gamma _m}{2} \left\langle {\hat{b}}\right\rangle -ig \left\langle {\hat{u}}_1\right\rangle \left\langle {\hat{u}}_2^\dagger \right\rangle -ig \alpha _1 \left\langle {\hat{u}}_2^\dagger \right\rangle , \end{aligned}$$

8 $$\begin{aligned} \frac{\partial \left\langle {\hat{u}}_1\right\rangle }{\partial t}= & {} -\frac{\Gamma _1}{2} \left\langle {\hat{u}}_1\right\rangle -ig^*\left\langle {\hat{b}}\right\rangle \left\langle {\hat{u}}_2\right\rangle , \end{aligned}$$

9 $$\begin{aligned} \frac{\partial \left\langle {\hat{u}}_{2}\right\rangle }{\partial t}= & {} -\frac{\Gamma _2}{2} \left\langle {\hat{u}}_{2}\right\rangle -ig\left\langle {\hat{u}}_{1}\right\rangle \left\langle {\hat{b}}^\dagger \right\rangle -ig \alpha _1 \left\langle {\hat{b}}^\dagger \right\rangle . \end{aligned}$$

It is worth mentioning that the average $\left\langle {\hat{u}}_1\right\rangle$ and $\alpha _1$ are essentially different. The average $\left\langle {\hat{u}}_1\right\rangle$ is time dependent and governed by the equation of motion (8). In contrary, $\alpha _1$ is an intensive classical quantity that is independent of time and subject only to dissipation. Our calculations show that $\left\langle {\hat{u}}_1\right\rangle$ tends to zero value at steady state for large $\alpha _1$ value. The rate equations for the fluctuations are given by:

10 $$\begin{aligned} \frac{\partial \delta {\hat{b}}}{\partial t}= & {} -\frac{\Gamma _m}{2} \delta {\hat{b}} -ig\left\langle {\hat{u}}_{2}^\dagger \right\rangle \delta {\hat{u}}_1 -ig\Big (\alpha _1+\left\langle {\hat{u}}_{1}\right\rangle \Big ) \delta {\hat{u}}_2^\dagger +\sqrt{\Gamma _m}{\hat{n}}_m, \end{aligned}$$

11 $$\begin{aligned} \frac{\partial \delta {\hat{u}}_1}{\partial t}= & {} -\frac{\Gamma _1}{2} \delta {\hat{u}}_1 -ig^*\left\langle {\hat{u}}_{2}\right\rangle \delta {\hat{b}} -ig^* \left\langle {\hat{b}}\right\rangle \delta {\hat{u}}_2+\sqrt{\Gamma _1}{\hat{n}}_1, \end{aligned}$$

12 $$\begin{aligned} \frac{\partial \delta {\hat{u}}_2}{\partial t}= & {} -\frac{\Gamma _2}{2} \delta {\hat{u}}_2 -ig\left\langle \hat{b^\dagger }\right\rangle \delta {\hat{u}}_1 -ig\Big (\alpha _1+\left\langle {\hat{u}}_{1}\right\rangle \Big ) \delta \hat{b^\dagger }+\sqrt{\Gamma _2}{\hat{n}}_2. \end{aligned}$$

The averages can be solved in the steady state for a given decay coefficient, i.e., $\Gamma _j$, and classical field component, i.e., $\alpha _1$. Thus, the fluctuations, i.e., $\delta {\hat{o}}$, can be solved in the frequency domain (as shown in “Methods” section).

According to the incident-reflected relation in a transmission line, ${\hat{b}}={\hat{b}}^{out}+{\hat{b}}^{in}$ for the case of total reflection. It then follows that the microwave output fluctuation is given by^34^:

13 $$\begin{aligned} \delta {\hat{b}}=\delta {\hat{b}}^{out}+{\hat{n}}_m, \end{aligned}$$

The optical output fluctuations $\delta {\hat{u}}_2^{out}$ and $\delta {\hat{b}}$ can be evaluated by solving Eqs. (7)–(12). The squeezing of the hybrid two-modes can be quantified by calculating its fluctuation correlation. The spectrum of this fluctuation correlation (named the squeezing spectrum) is given by:

14 $$\begin{aligned} S_X(\omega )=\left\langle \delta {\hat{X}}_{out}(\omega )\delta {\hat{X}}_{out}(\omega )\right\rangle , \end{aligned}$$

where $\delta {\hat{X}}_{out}(\omega )=\frac{1}{\sqrt{2}}\bigg (\delta {\hat{b}}^{out}(\omega )+\delta {\hat{u}}_2^{out}(\omega )+\delta {\hat{b}}^{out^\dagger }(\omega )+\delta {\hat{u}}_2^{out^\dagger }(\omega )\bigg )$^35^. Here, $\delta {\hat{o}}(\omega ) ={\mathcal {F}}(\delta {\hat{o}}(t))= \int _{-\infty }^{+\infty } \delta {\hat{o}}(t) e^{-i\omega t} \partial t$ is Fourier transform.

The squeezing spectrum is less than unity for two-mode squeezed quadrature^36,37^.

### Numerical estimations

In this section, numerical estimations are presented to study the viability of the proposed scheme. As detailed in Eq. (26), the squeezing spectrum is dependent on *g* (the coupling rate) and on $\Gamma _1$, $\Gamma _2$, and $\Gamma _m$ (the decay coefficients). Our numerical simulations show that squeezing is achieved for specific combination of decay coefficients and coupling rate. Upon setting $a=\frac{\pi c}{2 \omega _1 \sqrt{\varepsilon } }$, $\Gamma _1$ and $\Gamma _2$ slightly change with the microwave frequency (as the optical fields are off-resonant with the layered graphene medium). Thus, under this condition, squeezing can be extended to over a larger microwave frequency range by controlling the coupling rate against the microwave frequency. The coupling rate can be electrically modified by disturbing the effective electron density of graphene with a DC bias. Hereby, wideband microwave squeezing is achieved.

In the following simulations, we consider a cryogenic temperature $T=3$ mK and a silicon filling material with $\varepsilon =(3.5)^2$.

**Figure 3:**
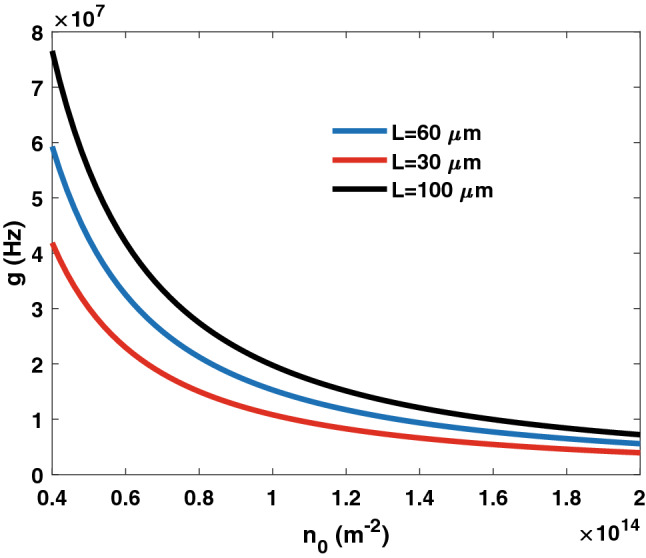
Coupling rate *g* versus graphene electron density. Several medium lengths are considered.

**Figure 4:**
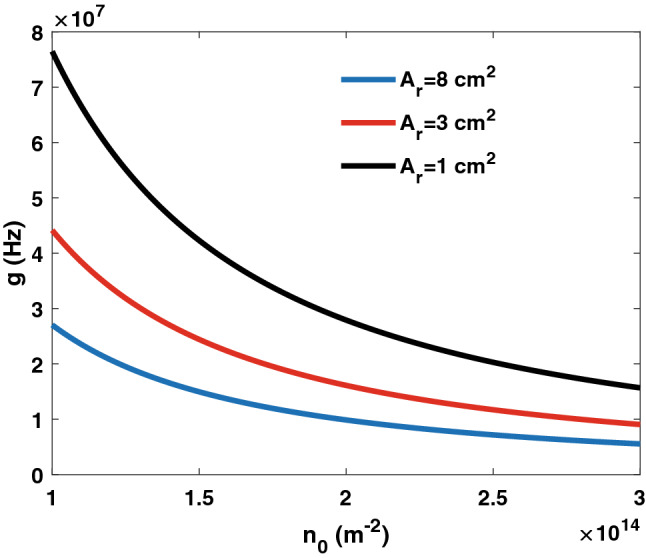
Coupling rate *g* versus graphene electron density. Different areas of the graphene layers are considered.

First, we evaluate the coupling rate versus different graphene design parameters. In Fig. 3, the coupling rate is evaluated versus the graphene electron density. Several medium lengths are considered. The coupling rate can be set over a wide range by controlling the electron density and the medium length (i.e., the number of layers). Moreover, in Fig. 4, the conversion rate versus the electron density is displayed considering different graphene cross-sectional areas. Figures 3 and 4 demonstrate the feasibility of controlling the coupling rate by altering the graphene layer properties.

**Figure 5:**
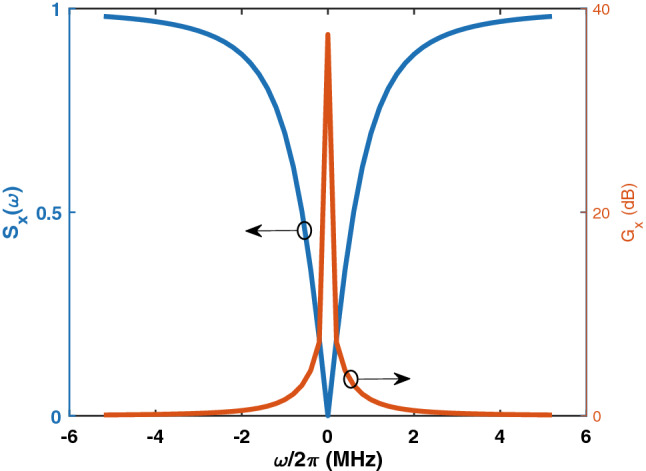
Squeezing spectrum $S_x(\omega )$ and squeezing gain $G_{X}$ of the output two-mode quadrature. Here, $\frac{\omega _m}{2\pi }=10$ GHz and $g=20.24 \times 10^{6}$ Hz.

**Figure 6:**
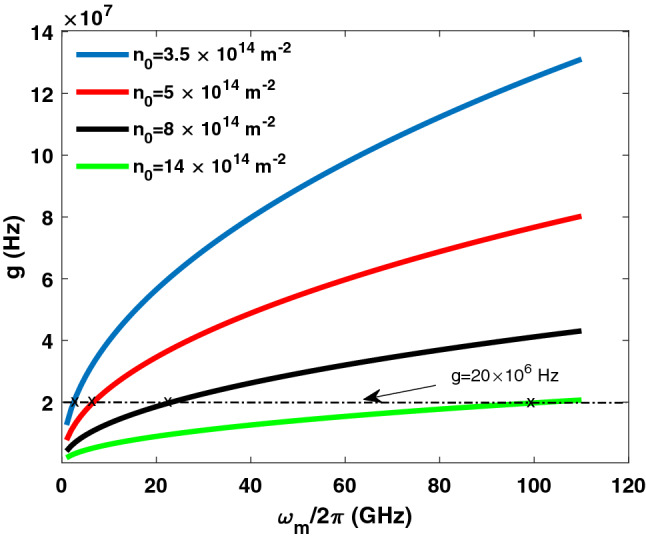
Coupling rate versus microwave frequency. Different electron densities are considered.

Upon finding the average values in the steady state, the squeezing spectrum can be evaluated. In Fig. 5, the squeezed spectrum $S_X$ and squeezing gain $\big ($defined by $G_{X}=-10log_{10}[S_X] \big )$ of the output two-mode quadrature are shown for $\frac{\omega _m}{2\pi }=10$ GHz. Here, $g=20.24 \times 10^{6}$ Hz, and $\alpha _1= 708$. The averages in the steady state are $\left\langle {\hat{b}}\right\rangle =200$, $\left\langle {\hat{u}}_1\right\rangle =0$, and $\left\langle {\hat{u}}_2\right\rangle =7$. The optical decay coefficients calculated from the dispersion relation are $\Gamma _1=\Gamma _2= 8\times 10^{11}$ Hz, and the microwave decay coefficient is $\Gamma _m=1.02\times 10^{9}$ Hz. These values were defined by carrying out significant numerical investigations. As seen, squeezing with significant gain is achieved over a bandwidth of almost 2 MHz with a peak of approximately 37 dB at $\omega =0$. Here, $\omega$ is the frequency spectrum of the fluctuations.

**Figure 7:**
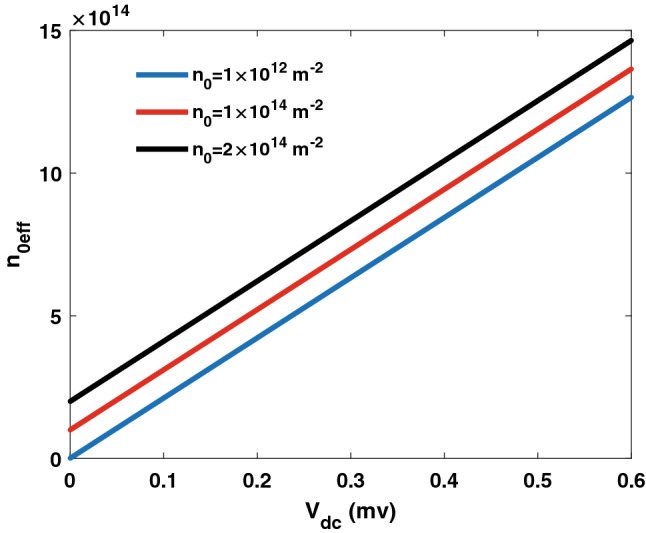
Effective electron density versus DC bias voltage. Different intrinsic electron densities are considered.

Our simulations show that by setting $a=\frac{\pi c}{2 \omega \sqrt{\varepsilon }}=0.11~\upmu{\text{m}}$, the decay coefficients change very slightly over the 10–100 GHz microwave frequency range. However, the coupling rate is function of the microwave frequency. Nonetheless, the coupling rate can be controlled by modifying the electron density of the graphene layers. It then follows squeezing can be extended against the microwave frequency range for proper combinations of $\omega _m$ and effective electron density. For instance, if the coupling rate is constant (against the microwave frequency) at $g=20.24 \times 10^{6}$ Hz, then the squeezing in Fig.  5 can be extended over the entire microwave frequency range from 10 to 100 GHz. In Fig. 6, the coupling rate is evaluated against the microwave frequency. Different electron densities are considered. As can be observed, the coupling rate can be maintained at $g=20.24 \times 10^{6}$ Hz over the entire microwave frequency range by adjusting the electron density from $3.5 \times 10^{14}~\text {m}^{-2}$ to $14 \times 10^{14}~\text {m}^{-2}$. The effective electron density can be modified by applying a DC electrical voltage as follows^38^:

15 $$\begin{aligned} n_{0_{eff}}=n_0+2\frac{C_T}{\pi q}V_{dc}, \end{aligned}$$

where $n_0$ is the intrinsic electron density and $V_{dc}$ is the DC bias voltage. The effective electron density is displayed in Fig. 7 against the DC bias voltage. As shown in Fig. 7, the effective electron density can be controlled over the required range, from $3.5 \times 10^{14}~\text {m}^{-2}$ to $14 \times 10^{14}~\text {m}^{-2}$, by varying the DC bias from 0.1 to 0.6 m V for intrinsic electron density $n_0=1 \times 10^{14}~\text {m}^{-2}$.

**Figure 8:**
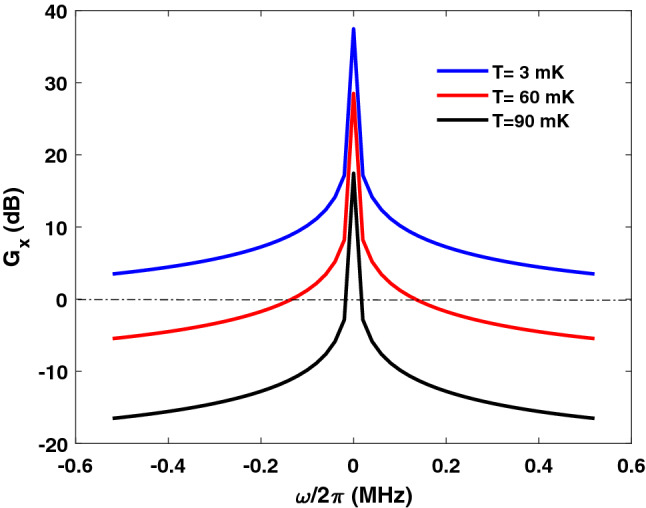
Squeezing gain spectrum. Different temperatures are considered.

Furthermore, we evaluate the squeezing gain against the temperature. In Fig. 8, the squeezing gain spectrum is evaluated for $T=3$ mK, $T=60$ mK, and $T=90$ mK. The other parameters are the same as in Fig. 5. We observe that the squeezing gain can be maintained even up to moderate cryogenic temperatures. For example, for $T=90$ mK, a squeezing gain can be obtained for an up to tens of kHz frequency bandwidth with a peak of 17 dB at $\omega =0$. Our numerical investigations show that the graphene conductivity slightly changes with the temperature over this range. However, the squeezing limitations are attributed to the thermal noise contribution. This is a further advantage of the proposed scheme since operations at moderate cryogenic temperatures is practical requirement for quantum processors^39^.

Finally, we evaluate the optical pump power needed to achieve squeezing, given by $p=\frac{1}{2}\varepsilon _0\sqrt{\varepsilon ^{\prime }} A_rA_{\alpha _1}$, where $A_{\alpha _1}=\big (\frac{\hbar \omega _1}{\varepsilon _0 \varepsilon ^{\prime } A_r L }\big )^\frac{1}{2}\alpha _1$ is the associated electric field intensity. For $\alpha _{1}=708$, the optical pump power is $p=577$ mWThis is a moderate optical power value that can be supported by an off-the-shelf optical source.

## Discussion

A novel modality for hybrid two-mode (microwave and optical) squeezing is proposed and thoroughly investigated utilizing graphene layered structure. The graphene layers are driven by a quantum electrical voltage and subjected to two optical fields. We have shown that by setting the frequency spacing between the optical fields equal to the microwave frequency and properly designing the classical component of the optical pump field, significant hybrid two-mode squeezing can be achieved. Inherited from the microwave photonics features, the proposed scheme exhibits several major advantages. First, the proposed scheme lays the ground to achieve hybrid quantum systems that leverage the advantages of both superconducting and photonic systems. Second, the structure is simple and requires only two optical fields with controlled frequency. Third, the microwave frequency can be simply tuned by controlling the optical frequency spacing and the coupling rate. Furthermore, the proposed scheme has a mild parametric temperature dependence, while the temperature limitations are attributed to the fluctuation–dissipation-type-induced thermal contribution. These properties open the way for practical implementation of squeezed hybrid modes in quantum microprocessors and other applications.

### Methods

For simplification, we consider the following fields transformations:

16 $$\begin{aligned} {\hat{u}}_1={\hat{U}}_1,~ {\hat{b}}=i{\hat{B}},~ {\hat{u}}_2={\hat{U}}_2 e^{i\Theta },~ g=\mid g\mid e^{i\Theta }. \end{aligned}$$

where $\Theta$ is a random phase angle. It then follows that the equations of motions are given by:

17 $$\begin{aligned} \frac{\partial {\hat{B}}}{\partial t}= & {} -\frac{\Gamma _m}{2} {\hat{B}}-\mid g\mid \big ({\hat{U}}_{1}+\alpha _1\big ){\hat{U}}_2^{\dagger }+\sqrt{\Gamma _m}{\hat{n}}_m, \end{aligned}$$

18 $$\begin{aligned} \frac{\partial {\hat{U}}_1}{\partial t}= & {} -\frac{\Gamma _1}{2} {\hat{U}}_{1}+ \mid g\mid {\hat{B}}{\hat{U}}_2+\sqrt{\Gamma _1}{\hat{n}}_1, \end{aligned}$$

19 $$\begin{aligned} \frac{\partial \alpha _1}{\partial t}= & {} -\frac{\Gamma _1}{2} \alpha _{1}, \end{aligned}$$

20 $$\begin{aligned} \frac{\partial {\hat{U}}_2}{\partial t}= & {} -\frac{\Gamma _2}{2} {\hat{U}}_{2}- \mid g\mid \big ({\hat{U}}_1+\alpha _1\big ){\hat{B}}^{\dagger }+\sqrt{\Gamma _2}{\hat{n}}_2, \end{aligned}$$

Similar to Eqs. (7)–(12), one can obtain rate equations for operators’ averages and fluctuations.

On considering steady state (where $$\frac{\partial \left\langle {\hat{O}}\right\rangle }{\partial t}=0$$), one gets 6 nonlinear equations of the 6 average operators: $$\left\langle {\hat{B}}\right\rangle $$, $$\left\langle {\hat{B}}^\dagger \right\rangle $$, $$\left\langle {\hat{U}}_1\right\rangle $$, $$\left\langle {\hat{U}}_{1}^\dagger \right\rangle $$$$\left\langle {\hat{U}}_2\right\rangle $$, and $$\left\langle {\hat{U}}_{2}^\dagger \right\rangle $$ . On having several mathematical substitutions, these 6 equations can be simplified in form of the following three equations:

21 $$\begin{aligned}&\frac{\Gamma _m \Gamma _2}{\mid g\mid ^2}\Bigg [1+\frac{4\mid g\mid ^2}{\Gamma _m \Gamma _1}\left\langle {\hat{U}}_{2}^\dagger \right\rangle \left\langle {\hat{U}}_{2}\right\rangle \Bigg ]^2 +16\frac{\mid g\mid ^2 \mid \alpha _1\mid ^2}{\Gamma _m \Gamma _1}\left\langle {\hat{U}}_{2}^\dagger \right\rangle \left\langle {\hat{U}}_{2}\right\rangle \nonumber \\&\quad =4\mid \alpha _1\mid ^2\Big (1+\frac{\mid g\mid ^2}{\Gamma _m \Gamma _1}\left\langle {\hat{U}}_{2}^\dagger \right\rangle \left\langle {\hat{U}}_{2}\right\rangle \Big ), \end{aligned}$$

22 $$\begin{aligned}&\left\langle {\hat{B}}\right\rangle =-2\alpha _1\frac{\mid g\mid }{\Gamma _m} \frac{\left\langle {\hat{U}}_{2}^\dagger \right\rangle }{1+\frac{4\mid g\mid ^2}{\Gamma _m \Gamma _1}\left\langle {\hat{U}}_{2}^\dagger \right\rangle \left\langle {\hat{U}}_{2}\right\rangle } \end{aligned}$$

23 $$\begin{aligned}&\left\langle {\hat{U}}_1\right\rangle =-4\alpha _1\frac{\mid g\mid ^2 }{\Gamma _m\Gamma _1} \frac{\left\langle {\hat{U}}_{2}^\dagger \right\rangle \left\langle {\hat{U}}_{2}\right\rangle }{1+\frac{4\mid g\mid ^2}{\Gamma _m \Gamma _1}\left\langle {\hat{U}}_{2}^\dagger \right\rangle \left\langle {\hat{U}}_{2}\right\rangle } \end{aligned}$$

The nonlinear Eq. (21) can be solved numerically, for known decay coefficients and classical pump, giving $\left\langle {\hat{U}}_{2}\right\rangle$ value. Consequently, $\left\langle {\hat{B}}\right\rangle$ and $\left\langle {\hat{U}}_{1}\right\rangle$ can be calculated using Eqs. (22) and (23).

Second, by applying the Fourier transform to the equation set of the fluctuations, the solution of the operator fluctuation, in the frequency domain, is given by:

24 $$\begin{aligned} {{\varvec{G}}}{{\varvec{R}}}={{\varvec{N}}}, \end{aligned}$$

where

25 $$\begin{aligned} {{\varvec{R}}}= & {} \begin{pmatrix} \delta {\hat{B}}(\omega )\\ \delta {\hat{B}}^\dagger (\omega )\\ \delta {\hat{U}}_1(\omega )\\ \delta {\hat{U}}_1^\dagger (\omega )\\ \delta {\hat{U}}_2(\omega )\\ \delta {\hat{U}}_2^\dagger (\omega ) \end{pmatrix}{,}\, {{\varvec{N}}}=\begin{pmatrix} \sqrt{\Gamma _m}{\hat{N}}_m(\omega )\\ \sqrt{\Gamma _m}{\hat{N}}_m^\dagger (\omega )\\ \sqrt{\Gamma _1}{\hat{N}}_1(\omega )\\ \sqrt{\Gamma _1}{\hat{N}}_1^\dagger (\omega )\\ \sqrt{\Gamma _2}{\hat{N}}_2(\omega )\\ \sqrt{\Gamma _2}{\hat{N}}_2^\dagger (\omega ) \end{pmatrix}, \end{aligned}$$

26 $$\begin{aligned} {{\varvec{G}}}= & {} \begin{pmatrix} i\omega +\frac{\Gamma _m}{2}&{} 0 &{} \mid g\mid \left\langle {\hat{U}}_{2}^\dagger \right\rangle &{} 0 &{} 0 &{} \mid g\mid \alpha _1+\mid g\mid \left\langle {\hat{U}}_{1}\right\rangle \\ 0 &{} -i\omega +\frac{\Gamma _m}{2} &{} 0 &{} \mid g\mid \left\langle {\hat{U}}_{2}\right\rangle &{} \mid g\mid \alpha _1^*+\mid g\mid \left\langle {\hat{U}}_{1}^\dagger \right\rangle &{} 0 \\ -\mid g\mid \left\langle {\hat{U}}_{2}\right\rangle &{} 0 &{} i\omega +\frac{\Gamma _1}{2} &{} 0 &{} -\mid g\mid \left\langle {\hat{B}}\right\rangle &{} 0 \\ 0 &{} -\mid g\mid \left\langle {\hat{U}}_{2}^\dagger \right\rangle &{} 0 &{} -i\omega +\frac{\Gamma _1}{2} &{} 0 &{} -\mid g\mid \left\langle {\hat{B}}^\dagger \right\rangle \\ 0 &{} \mid g\mid \alpha _1+\mid g\mid \left\langle {\hat{U}}_{1}\right\rangle &{} \mid g\mid \left\langle {\hat{B}}^\dagger \right\rangle &{} 0 &{} i\omega +\frac{\Gamma _2}{2} &{} 0 \\ \mid g\mid \alpha _1^*+\mid g\mid \left\langle {\hat{U}}_{1}^\dagger \right\rangle &{} 0 &{} 0 &{} \mid g\mid \left\langle {\hat{B}}\right\rangle &{} 0 &{} -i\omega +\frac{\Gamma _2}{2} \end{pmatrix}. \end{aligned}$$

For instance, the solution for the microwave annihilation operator is given by:

27 $$\begin{aligned} \begin{aligned} \delta {\hat{B}}(\omega )=&T_{1,1} \sqrt{\Gamma _m}{\hat{N}}_m(\omega )+T_{1,2} \sqrt{\Gamma _m}{\hat{N}}_m^\dagger (\omega )+ T_{1,3} \sqrt{\Gamma _1}{\hat{N}}_1(\omega )\\&+ T_{1,4} \sqrt{\Gamma _1}{\hat{N}}_1^\dagger (\omega )+T_{1,5} +\sqrt{\Gamma _2}{\hat{N}}_2(\omega )+T_{1,6} \sqrt{\Gamma _2}{\hat{N}}_2^\dagger (\omega ), \end{aligned} \end{aligned}$$

where $T_{l,k}=G^{-1}(l,k)$, $\langle N(\omega _1)^\dagger N(\omega _2) \rangle = \frac{2\pi }{exp(\hbar \omega /k_BT)-1} \delta (\omega _1+\omega _2)$, and $\langle N(\omega _1) N(\omega _2)^\dagger \rangle = 2\pi \ + \langle N(\omega _1)^\dagger N(\omega _2) \rangle$. The solution for the other operators can be obtained in a similar way.

## Supplementary information

Supplementary information.

## Data Availability

The datasets generated during and/or analysed during the current study are available from the corresponding author on reasonable request.
