# Supplementary material for: Hybrid two-mode squeezing of microwave and optical fields using optically pumped graphene layers
Source: Sci Rep. 2020 Oct 7;10:16676. doi: 10.1038/s41598-020-73363-y (PMC7541456; doi:10.1038/s41598-020-73363-y)
Supplement: Supplementary file 1 — Supplementary information. [file 41598_2020_73363_MOESM1_ESM.pdf]

Supplementary Material

This is a supplementary document providing details on the derivation of the quantum Hamiltonian of the proposed system in the paper.

1. CLASSICAL FIELDS

From classical point of view, the graphene layers are electrically driven by microwave signal $V_m = ve^{-i\omega_m t} + c.c.$, and subjected to two optical waves with associated electric fields $\vec{E}_j = A_j e^{-i\left(\omega_j t - \beta_j z\right)} \vec{e_y} + c.c.$. Here, $j \in \{1, 2\}$.

On obeying the same approach reported in our previous work[1], the following steps are carried out :

First, the effective permittivity of the graphene layers is approximated by implementing a perturbation approach, yielding:

$$\varepsilon_{eff} = \varepsilon'_{eff} + \left(v\varepsilon''_{eff} e^{-i\omega_m t} + c.c.\right), \tag{S1}$$

where ε'_{eff} and ε''_{eff} are the unperturbed and the perturbed permittivity terms defined in the main text of the paper. Second, the classical Hamiltonian is obtained, using the derived effective permittivity, reading:

$$\mathcal{H} = \varepsilon_0 \varepsilon'_{eff} \left(|A_1|^2 + |A_2|^2\right) V_{ol} + 2\varepsilon_0 \varepsilon''_{eff} A_1 A_2^* v^* sinc\left(\frac{\beta_1 + \beta_2}{2} L\right) e^{i\frac{\beta_1+\beta_2}{2} L} V_{ol} + c.c., \tag{S2}$$

where $V_{vol} = A_r \times L$ is the volume occupied by the graphene layers, and $c.c.$ stands for complex conjugate.

2. FIELDS QUANTIZATION

The microwave and optical fields can be quantized using the relations:

$$A_j = \left(\frac{\hbar\omega_j}{\varepsilon'_{eff}\varepsilon_0 V_{vol}}\right)^{\frac{1}{2}} \hat{u}_j, \quad v = \left(\frac{\hbar\omega_m}{CA_r}\right)^{\frac{1}{2}} \hat{b}. \tag{S3}$$

where $\hat{b}$ and $\hat{u}_j$ are the annihilation operators of the microwave and optical fields, respectively. Finally, the quantum Hamiltonian $\hat{\mathcal{H}} = \hat{\mathcal{H}}_0 + \hat{\mathcal{H}}_1$ is obtained by substituting the quantization relation in (S3) into the Hamiltonian expression in (S2), yielding:

$$\hat{\mathcal{H}}_0 = \hbar\omega_m \hat{b}^\dagger \hat{b} + \sum_{j=1}^{2} \hbar\omega_j \hat{u}_j^\dagger \hat{u}_j, \tag{S4}$$

$$\hat{\mathcal{H}}_1 = \hbar g \hat{u}_2^\dagger \hat{b}^\dagger \hat{u}_1 + \hbar g^* \hat{u}_1^\dagger \hat{b} \hat{u}_2 + h.c., \tag{S5}$$

where g is the coupling rate defined in the main text of the paper, and $h.c.$ stands for the hermitian conjugate .

REFERENCES

1. M. Qasymeh and H. Eleuch, "Quantum microwave-to-optical conversion in electrically driven multilayer graphene," Opt. Express **27**, 5945–5960 (2019).
